# Properdin, transcortin and HGFAC are novel plasma biomarkers in canine chronic inflammatory enteropathies from active disease to remission

**DOI:** 10.1038/s41598-025-11474-0

**Published:** 2025-07-15

**Authors:** Pavlos G. Doulidis, Carolina Frizzo Ramos, Alexandro Rodriguez-Rojas, Franziska Roth-Walter, Iwan A. Burgener

**Affiliations:** 1 Division of Small Animal Internal Medicine, Department for Companion Animals and Horses, University of Veterinary Medicine Vienna, Veterinärplatz 1, Vienna, 1210 Austria; 2 Department of Biological Sciences and Pathobiology, University of Veterinary Medicine Vienna, Veterinärplatz 1, Vienna, A-1210 Austria

**Keywords:** Inflammatory bowel disease, Pathogenesis

## Abstract

Canine chronic inflammatory enteropathies (CCIE) is a group of intestinal inflammatory conditions causing chronic or relapsing gastrointestinal symptoms. Accurate diagnosis, treatment and monitoring remain challenging, necessitating novel diagnostic tools. This study aims to investigate the plasma proteome of ten dogs with histologically confirmed CCIE during active disease and clinical remission compared to ten healthy controls. We utilized surplus lithium-heparin plasma and performed label-free quantification mass spectrometry. A significant upregulation of complement factor properdin (CFP) during disease was noted, pointing toward microbial-driven intestinal inflammation. During remission, CFP levels remained elevated compared to controls, indicating persistent subclinical inflammation. We report hepatocyte growth factor activator (HGFAC) as a novel canine plasma protein associated with decreased risk for CCIE and as a potential therapeutic target, similarly, as reported in humans. Linear regression analysis revealed that disease severity was negatively correlated to transcortin/SERPINA6, as negative acute phase protein. Proteins involved in inflammation and tissue repair, such as inter-alpha-trypsin inhibitor heavy chain 4, (ITIH4), and anti-inflammatory molecules like apolipoprotein A-IV (APOA4), were significantly upregulated in remission. Conversely, proteins related to DNA remodeling and methylation, including histone H2B and carboxypeptidase N subunit 2 (CPN2), were downregulated during remission. Gene ontology analysis revealed altered pathways linked to immune response and coagulation. In CCIE patients we identified for the first time markers such as properdin for intestinal inflammation, while transcortin and HGFAC may serve as markers for remission. Future studies with larger cohorts are needed to validate the use of these proteins for monitoring disease progression and remission and refine their clinical applicability.

**Supplementary Information:**

The online version contains supplementary material available at 10.1038/s41598-025-11474-0.

## Introduction

Canine chronic inflammatory enteropathies (CCIE) are one of the most common causes of chronic or relapsing gastrointestinal (GI) symptoms in dogs, manifesting with a variety of symptoms like diarrhea, vomitus, anorexia and weight loss that last longer than 3 weeks^1,2^. The exact mechanism of CIE is still unclear but includes, similar to human inflammatory bowel disease (IBD), genetic, environmental and nutritional factors, as well as fecal microbiome composition and intestinal immune response^3–5^. Therefore, the terms CCIE and canine IBD have been interchangeably used to describe the same pathological process. Depending on the response to treatment and clinical improvement, CCIE can be classified as food responsive enteropathy (FRE), antibiotic responsive enteropathy (ARE), immunosuppression responsive enteropathy (IRE) and non-responsive enteropathy (NRE)^6–8^. Diagnosing CCIE can be challenging and requires a thorough history, clinical examination, exclusion of systemic, endocrine, neoplastic and infectious causes of chronic GI signs and a histologic evidence of chronic intestinal inflammatory process in endoscopically or surgically obtained specimens^7,9^. Despite the many differences in histological patterns, histopathological examination usually reveals intestinal inflammatory cell infiltration and mucosal damage in both humans and dogs, underlying the similarities between the two species^10^. Even though histopathology is at the moment the gold standard for diagnosing and histologically classifying CCIE, significant interobserver variabilities have been reported^9,11^ and often histological findings do not correlate with clinical presentation or response to treatment^6^.

In the last years label free quantification liquid chromatography mass spectrometric (LC-MS) analysis of body fluids and tissues has gained exponential attention, as it offers high specificity for protein detection and quantification due to the uniqueness of peptide masses and sequences, in contrast to colorimetric enzyme tests and immunoassays^12,13^. Proteomic analysis has found use in many areas of veterinary research and provides valuable information that can contribute in the better understanding and even diagnosis of some diseases^14–22^. As routine biomarkers offer low sensitivity and specificity in diagnosing and classifying CCIE, LC-MS can be a promising tool for the discovery of novel biomarkers^23,24^.

Accordingly, proteomic analysis has been utilized in the area of gastroenterology to promote the discovery of novel biomarkers and protein alterations in people with chronic GI disease^25–27^. In dogs with CCIE, recent studies have established fecal proteins that can be used to diagnose chronic intestinal inflammation and protein loss like calprotectin and α1-proteinase inhibitor, but also investigated the whole fecal proteome^28–30^. By examining the protein expression profiles on canine intestinal organoids we have previously uncovered protein expression patterns that mirror those observed in human IBD, underscoring shared pathogenic pathways^31^. Fewer studies have evaluated the serum proteome or amino acid profile of dogs with chronic enteropathy (CE)^32–34^, but to the best of our knowledge no study has reported the plasma proteome signatures of CCIE during disease and after reaching clinical remission. Plasma has the advantage of including important factors of the coagulation cascade that could demonstrate significant pattern alterations during acute and chronic disease^35,36^. In a previous study, we have established a methodology to optimize protein detection in the canine plasma^37^. After reporting the signatures of inflammatory enteropathy in the microbiome^38^ as well as fecal and blood metabolome^39,40^ of a breed-specific cohort with characteristic CCIE, we aim to report and compare the plasma proteome of dogs suffering from CCIE during clinical disease and after entering clinical remission to each other and to the plasma proteome of a healthy control population. We hypothesize that dogs with CCIE have several up- and down-regulated plasma proteins during disease as well as during remission. Furthermore, we hypothesize that the plasma proteome of dogs suffering from CCIE differs from healthy dogs, even after entering remission, similarly to what we reported for the fecal microbiome of dogs with breed-specific CCIE^38^.

## Materials and methods

### Animals, groups and sample collection

#### CCIE group

Client-owned dogs with chronic (≥ 3-week duration) or intermittent (chronic signs with symptom-free intervals) GI signs (diarrhea, vomiting, weight loss, anorexia) or pleural or abdominal effusion (transudate), presented to the Clinic for Small Animal Internal Medicine of the University of Veterinary Medicine Vienna, Austria, over a 12-month period (September 2023 and September 2024) were enrolled in this study as the CCIE group. All methods were carried out in accordance with relevant Austrian guidelines. Wherever applicable, experimental protocols were approved by the Ethic commission of the Veterinary Medicine University Vienna. After history taking, thorough routine diagnostic testing was performed and included a physical exam, CBC, serum biochemical profile, C-Reactive Protein (CRP), measurement of bile acids and basal cortisol concentrations, ACTH-stimulation test (if basal cortisol < 2 µg/dl), and the assessment of serum concentration of cTLI (canine trypsin-like- immunoreactivity), SpecPL (specific pancreatic lipase), and cobalamin. Surplus lithium heparin plasma from the routine blood collection upon initial examination was used for LC-MS. Additionally, urinalysis for urine protein creatinine (UPC) ratio and urinary sediment, abdominal ultrasonography, and analysis of fecal samples by flotation and fecal Giardia antigen test were performed. After exclusion of other possible causes of chronic GI symptoms and effusions, and after completion of an elimination diet trial with hydrolyzed diet for at least four weeks in stable patients, all ten patients underwent gastroduodenoscopy by one of the authors (PGD). Patients with severe disease (e.g. severe hypoproteinemia, hypalbuminemia, ascites) underwent gastroduodenoscopy sooner to ensure prompt treatment. Diagnosis and histologic classification of CCIE was made by a board certified pathologist after histopathologic evidence of the characteristic intestinal inflammation pattern, based on the published guidelines^41^. The canine chronic enteropathy activity index (CCECAI) was used on enrolment and resampling day to calculate clinical severity in all dogs^42^. This scoring is based on severity alterations in nine different relevant criteria. These criteria are attitude and activity, appetite, vomiting, fecal consistency, defecation frequency, weight loss, serum albumin concentration, peripheral edema and ascites, and pruritus; each scored on a scale from 0 to 3. After summation, the total score is determined to be clinically insignificant (0–3), mild (4–5), moderate (6–8), severe (9–11), or very severe (≥ 12) CCIE. Dogs from the CCIE group continued receiving a hydrolyzed diet and depending on disease severity and histopathological findings, prednisolone was prescribed and was tapered down in a 2-week base until discontinuation, based on clinical response. In case of serious, life-threatening disease (e.g. severe hypoproteinemia, hypalbuminemia, severe hypercoagulability, not well tolerated glucocorticoid treatment in the past), cyclosporine A was added to the treatment to ensure more effective immunosuppression. In case of hypocobalaminemia or low serum folate concentration, supplementation was prescribed. After reaching remission these dogs were considered as the Remission group (RG). Clinical remission was defined by a decrease of CCECAI scores to ≤ 3. Finally, the earliest 50 days after treatment initiation, individuals in the group were revaluated, and blood sampling was repeated.

#### Control group

For the control group we used surplus plasma of clinically healthy client-owned dogs (*N* = 10) with no signs of disease within the last two months who were presented at the Clinic for Small Animal Internal Medicine (Veterinary University of Vienna, Vienna, Austria) over a period of six months for clinical examination and blood sampling. They were enrolled in a behavioral study (Ref: BMBWF 20221 − 0.210.26) conducted by the Interuniversity Messerli Institute of Research (Veterinary University of Vienna, Austria). All experimental protocols were approved by the Ethic commission of the Veterinary Medicine University Vienna. Prior to enrollment, written informed consent was obtained from the owners. Dogs of different breeds, body weights, and genders, ranging in age from 1 to 10 years old, were considered for the proteomics study. Inclusion criteria involved a comprehensive evaluation, including a detailed history, physical examination, and blood sampling performed by two authors (PGD, CFR). Complete blood count, serum or plasma biochemical profile, and electrolyte measurements were conducted. Additionally, the body condition score (Nestle Purina scale: ranging from 1-very thin to 9-significant obesity) of each dog was recorded. Dogs younger than one year, weighing less than 5 kg, and those with any pathological signs or recent medication administration, like antibiotics or glucocorticoids, within the last two months were excluded from the study. Likewise, dogs with significant alterations in any blood parameters, that could suggest comorbidities or severe complications were not enrolled. Due to ethical concerns, gastroduodenoscopy was not performed in control dogs, and not repeated in dogs in clinical remission. All methods were conducted in accordance with relevant Austrian guidelines and regulations and the ARRIVE guidelines (https://arriveguidelines.org) were followed.

Quantitative proteome analysis by label-free liquid chromatography–mass spectrometry (LC–MS).

Initial blood samples were collected using 2 ml Vacuette tubes with lithium heparin 13 × 75 green cap-white ring PREMIUM (Greiner Bio-One GmbH, Bad Haller Str. 32, 4550, Upper Austria, Austria). After centrifugation at 2000 x g for 5 min, the plasma was separated and stored at −21 °C. Surplus lithium heparin plasma was used for this study. Five µl of plasma from each of the 20 individuals was collected by centrifugation and was transferred to a tube containing 20 µl of urea denaturing buffer (6 M urea, 2 M thiourea, and 10 mM HEPES; pH 8.0). Disulfide bonds from the plasma proteins were reduced by adding 1 µl of dithiothreitol (10 mM) and incubated for 30 min at room temperature. Afterwards, the samples were alkylated by adding 1 µl of iodoacetamide (55 mM) solution and incubated at room temperature for another 30 min in the dark. The samples were diluted with four volumes of ammonium bicarbonate buffer (40 mM) and digested overnight at 37 °C by adding 1 µl of trypsin protease (Thermo Fischer Scientific, USA) (1 µg/µl). To acidify the samples, 5% acetonitrile and 0.3% trifluoroacetic acid (TFA; final concentration) were added, and subsequently, the samples were desalted using C18 StageTips with Empore™ C18 Extraction Disks^43^. The peptides eluted from the StageTips were dried using vacuum centrifugation.

Peptides were reconstituted in 20 µl of a solution containing 0.05% TFA and 4% acetonitrile. Then, 1 µl of each sample was applied to an Ultimate 3000 reversed-phase capillary nano liquid chromatography system connected to a Q Exactive HF mass spectrometer (Thermo Fisher Scientific). The samples were injected and concentrated on a PepMap100 C18 trap column (3 μm, 100 Å, 75 μm inner diameter [i.d.] × 20 mm, nanoViper; Thermo Fischer Scientific) that was equilibrated with 0.05% TFA in water. After switching the trap column inline, LC separations were performed on an Acclaim PepMap100 C18 capillary column (2 μm, 100 Å, 75 μm i.d. × 250 mm, nanoViper; Thermo Fischer Scientific) at an eluent flow rate of 300 nl/min. Mobile phase A consisted of 0.1% (v/v) formic acid in water, while mobile phase B contained 0.1% (v/v) formic acid and 80% (v/v) acetonitrile in water. The column was pre-equilibrated with 5% mobile phase B, followed by an increase to 44% mobile phase B over 100 min. Mass spectra were acquired in a data-dependent mode, utilizing a single MS survey scan (m/z 350–1650) with a resolution of 60,000, and MS/MS scans of the 15 most intense precursor ions with a resolution of 15,000. The dynamic exclusion time was set to 20 s, and the automatic gain control was set to 3 × 106 and 1 × 105 for MS and MS/MS scans, respectively.

MS and MS/MS raw data analysis was performed using the MaxQuant software package (version 2.6.6.0) with the implemented Andromeda peptide search engine. The data were searched against the Canis lupus familiaris reference proteome (ID: UP000805418; downloaded from Uniprot.org on 20.09.2024; 43,623 sequences) using the default parameters and enabling the options of label-free quantification (LFQ) and match between runs^44^. Data filtering and statistical analysis were conducted using the Perseus 2.1.3.0 software^45^. Proteins that were identified and quantified with LFQ intensity values in at least three (out of ten) replicates within at least one of the three experimental groups were used for downstream analysis. The rest were excluded. Missing values were imputed from a normal distribution using the default settings (width 0.3, downshift 1.8). Student’s t-test was used to calculate mean log2-fold differences between groups in Perseus. Proteins with a minimum 2-fold intensity change compared to the control (log2-fold change ≥ 1 or log2-fold change ≤ −1) and a p-value ≤ 0.05 were considered significantly abundant. All statistical comparisons between groups were performed using student’s t-Test implemented by the Perseus computational platform^45^. Adjusted P-values after FDR (q-values) were considered significant for values below 0.05. Statistical analysis and plotting were performed with the R 4.4.2 program. Normality was tested using the Shapiro-Wilk test for normality. Not normally distributed parameters were compared with the Mann-Whitney U test, while for normally distributed values the ANOVA test was used. Correlation testing was performed using Pearsons’s test, followed by regression analysis.

## Results

### Animals and groups

#### CCIE group and remission group

**Fig. 1:**
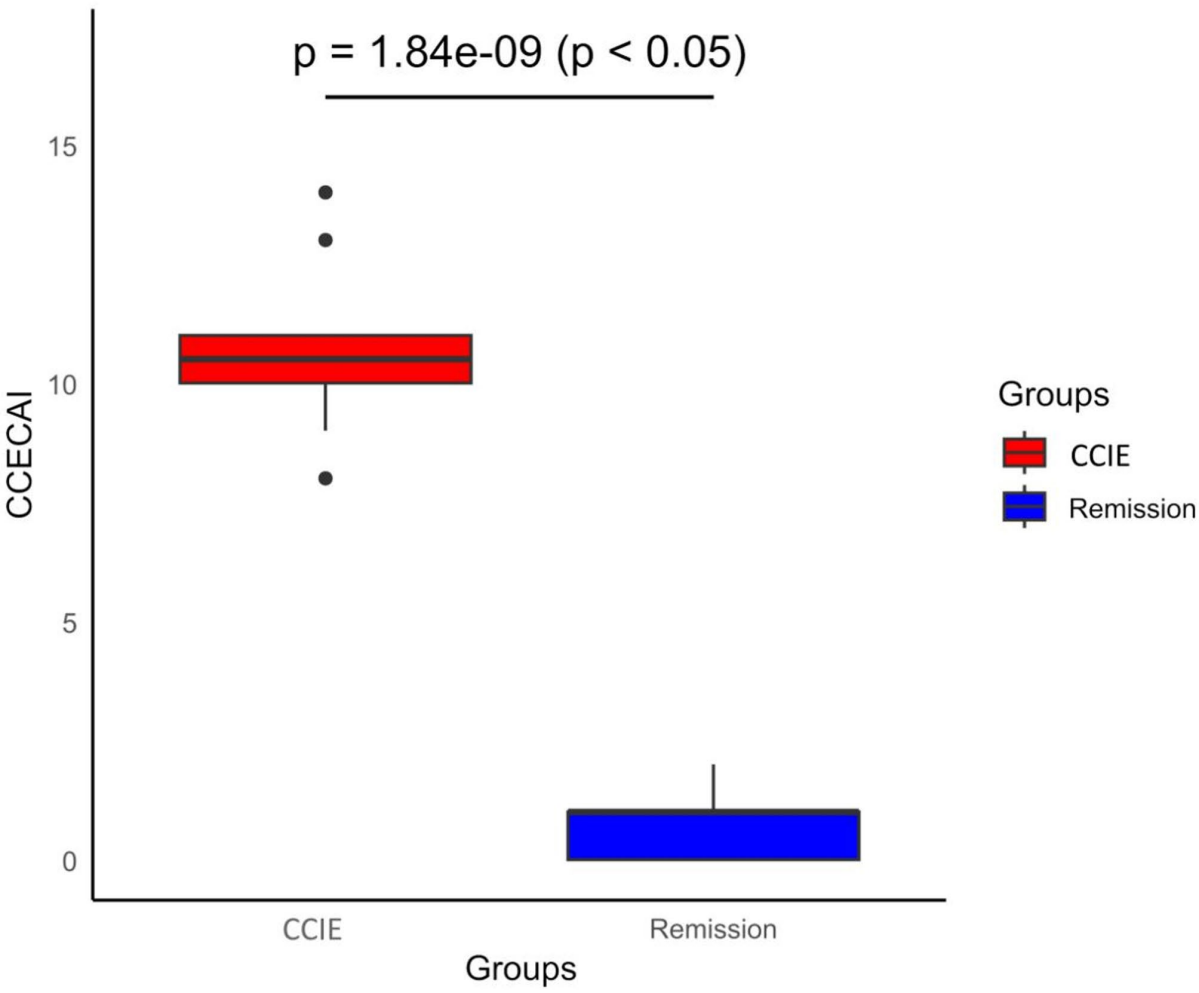
Boxplot demonstrating the statistically significant difference in the canine chronic enteropathy activity index (CCECAI) of the dogs in the CCIE patients upon enrollment and after reaching clinical remission.

**Fig. 2:**
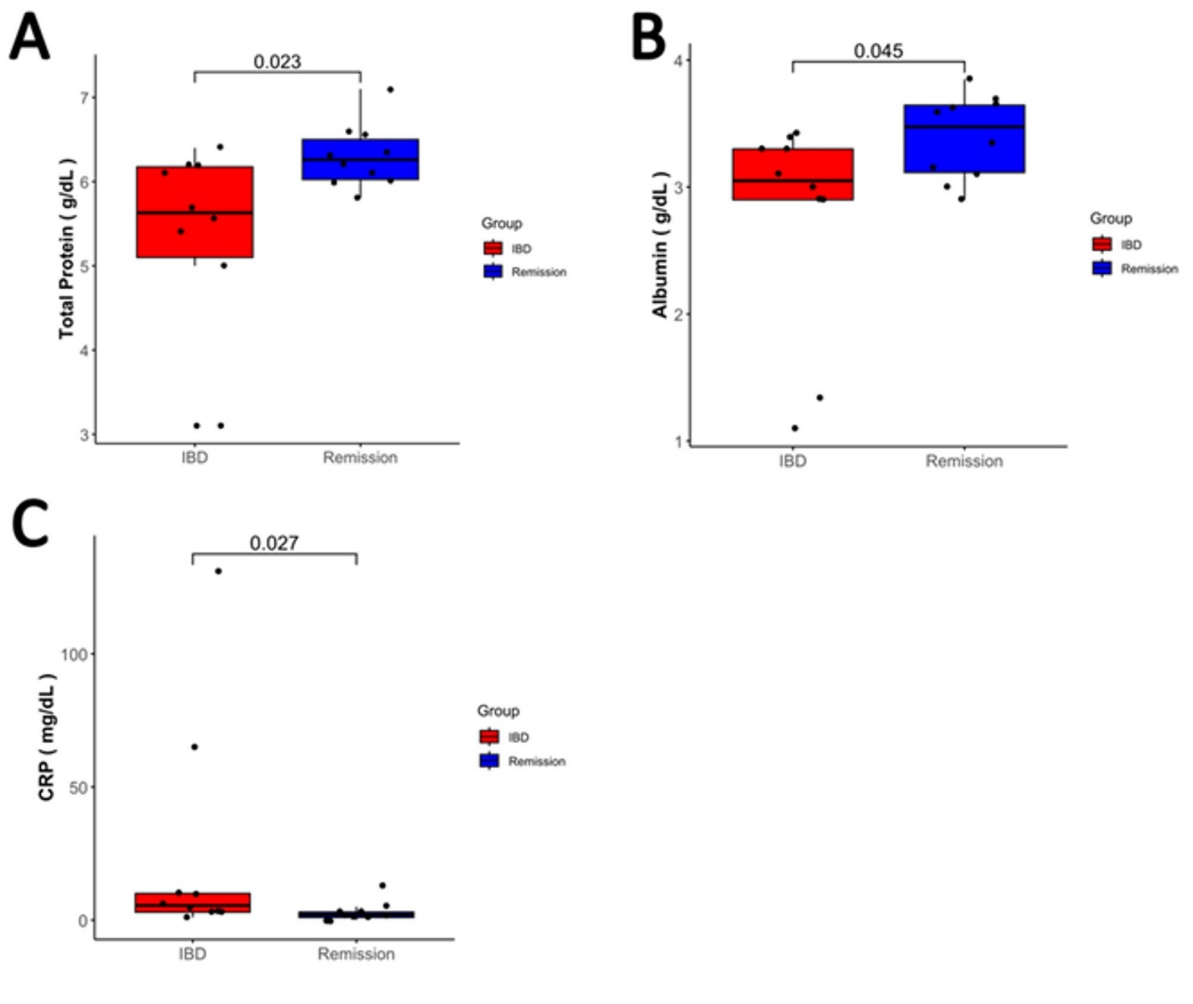
Boxplots illustrating the statistically significant differences in the clinicopathological parameters of active disease (CCIE) and remission.

The CCIE group was comprised of 10 dogs. All 10 dogs completed the study and entered the RG. 60% (60% *N* = 6) were male and 40% (*N* = 4) were female. Median age was 2,5 years old (range 1–12 years old). Median weight of the dogs was 19.7 kg (range 2.2–55 kg), while the same dogs during clinical remission had a median weight of 21.55 kg (range 2.5–58 kg). Breeds included Yorkshire Terrier (2), German Shepherd Dog (1), Malteser (1), Australian Shepherd (1), Welsh Terrier (1), Saint Bernard (1), Irish Wolfhound (1), Entlebucher Sight Dog (1), Mix breed (1). All ten dogs (*N* = 10, 100%) had a diagnosis of chronic lymphoplasmacellular inflammatory enteropathy according to WSAVA guidelines with no signs of lymphangiectasia in the examined duodenal samples. Of these dogs 5 responded to hydrolyzed diet alone (food responsive, *N* = 5, 50%), 4 (*N* = 4, 40%) received prednisolone and 1 dog with severe protein losing enteropathy (PLE) (*N* = 1, 10%) received prednisolone and cyclosporine A (CsA). These five dogs were classified as immunosuppression responsive. All of these 5 patients with IRE underwent unsuccessful food trials by the primary care veterinarian before but were also prescribed and kept on hydrolyzed diet. Prednisolone was initially prescribed at a dosage of 1 mg/kg twice daily and was tapered down on a biweekly base and for the dog with PLE, CsA was prescribed at a dosage of 5 mg/kg twice daily. The median CCECAI score of the CCIE group was 10.1 (range 8–14) and the median CCECAI of the same dogs in the RG was 1 (range 0–3). The difference between the two groups was statistically significant (*p* < 0.0001) (Fig. 1). All dogs achieved clinical remission (*N* = 10, 100%). Median time to remission reported and resampling was 71 days (range 35–171 days). In the 4 dogs that received prednisolone, medication was discontinued after 6–8 weeks, while in patient receiving prednisolone and CsA, prednisolone was discontinued after 6 weeks and CsA was reduced to once daily as maintenance treatment. Significant changes in hematology and serum biochemistry between active disease and remission were noticed only in serum total protein, albumin, CRP, and cobalamin although a significant outlier (PLE dog) influenced the CRP analysis. These changes are illustrated in Fig. 2. More specifically, albumin (CCIE median and range 3.05 g/dl (1.13 g/dl-3.43 g/dl) vs. RG median and range 3.48 g/dl (2.9 g/dl to 3.85 g/dl, *p* = 0.045) and total protein (CCIE median and range 5.63 g/dl (3.1 g/dl-6.4 g/dl) vs. RG median and range 6.26 g/dl (5.8 g/dl to 7.1 g/dl), *p* = 0.023) were significantly lower in the CCIE group, as expected due to the presence of dogs with protein losing enteropathy and panhypoproteinemia. CRP was significantly higher in the CCIE group, but with the presence of an outlier. Cobalamin was significantly higher after supplementation and achievement of clinical remission group (median 906pg/dL (range 460-1001pg/dL) compared to before treatment (median 260pg/dL (range 150-417pg/dL)).

#### Control group

Ten dogs were assigned to the control group. 50% (50%, *N* = 5) were male and 50% (*N* = 5) were female. Median age was 5,2 years old (range 2–9 years old) and median weight was 19,95 kg (range 9.8–34,8 kg). Breeds in the control group included mix breed (*N* = 5), Australian Shepherd (*N* = 2), German Shepherd (*N* = 1), Labrador Retriever (*N* = 1), Golden Retriever (*N* = 1).

##### Quantitative plasma proteome analysis by LC-MS

**Fig. 3:**
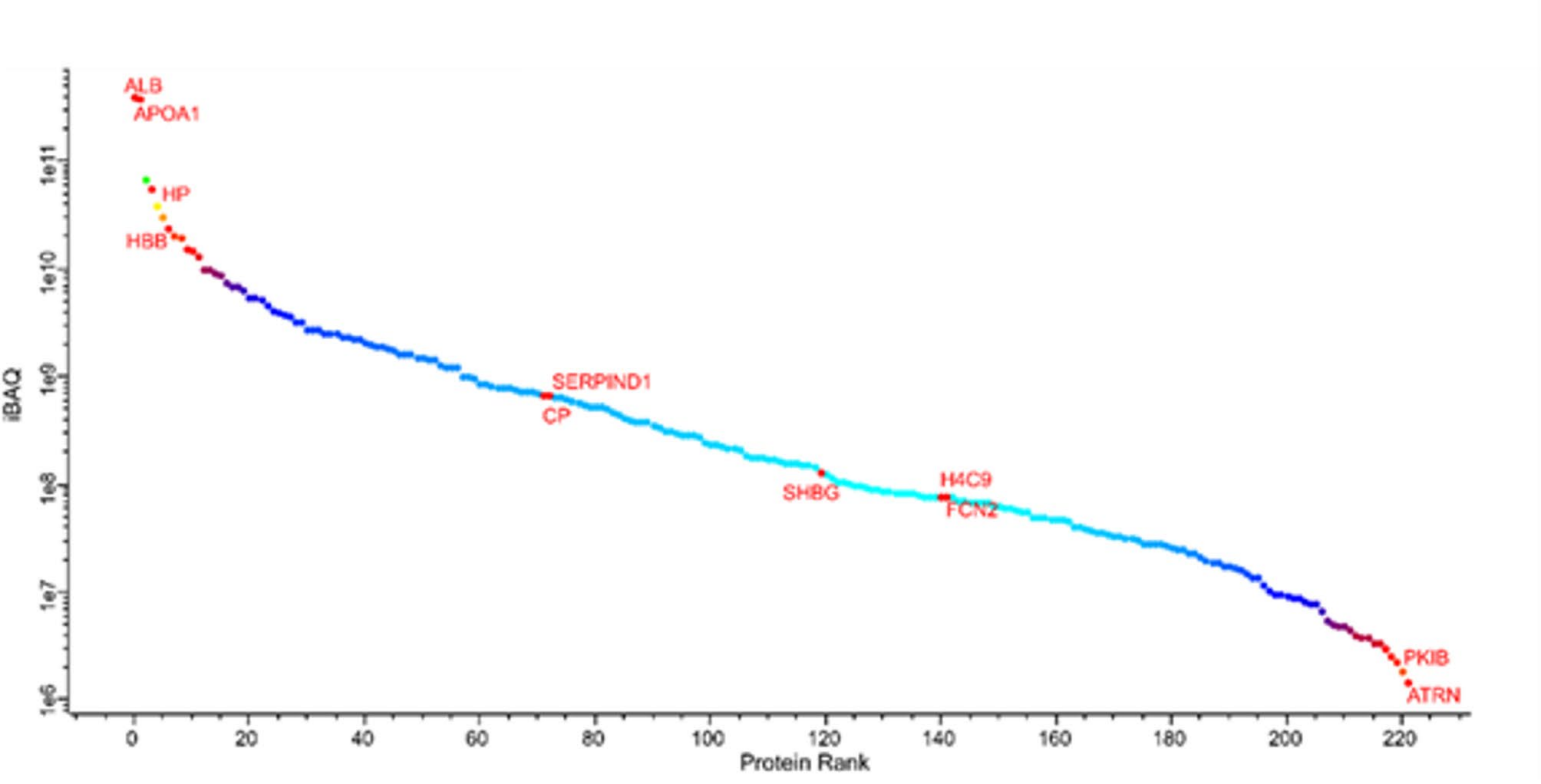
Abundance rank of the identified canine plasma proteins amongst all samples (iBAQ). Different levels of abundances are represented by the labelled proteins. ALB = Albumin, APOA1 = Apolipoprotein 1, HP = Haptoglobin, HBB = Hemoglobin subunit beta, SERPIND1 = Plasminogen activator inhibitor-1, CP = Ceruloplasmin, SHBG = Sex hormone-binding globulin, H4C9 = H4 Clustered Histone 9, FCN2 = Ficolin 2, PKIB = CAMP-Dependent Protein Kinase Inhibitor Beta, ATRN = Attractin.

**Fig. 4:**
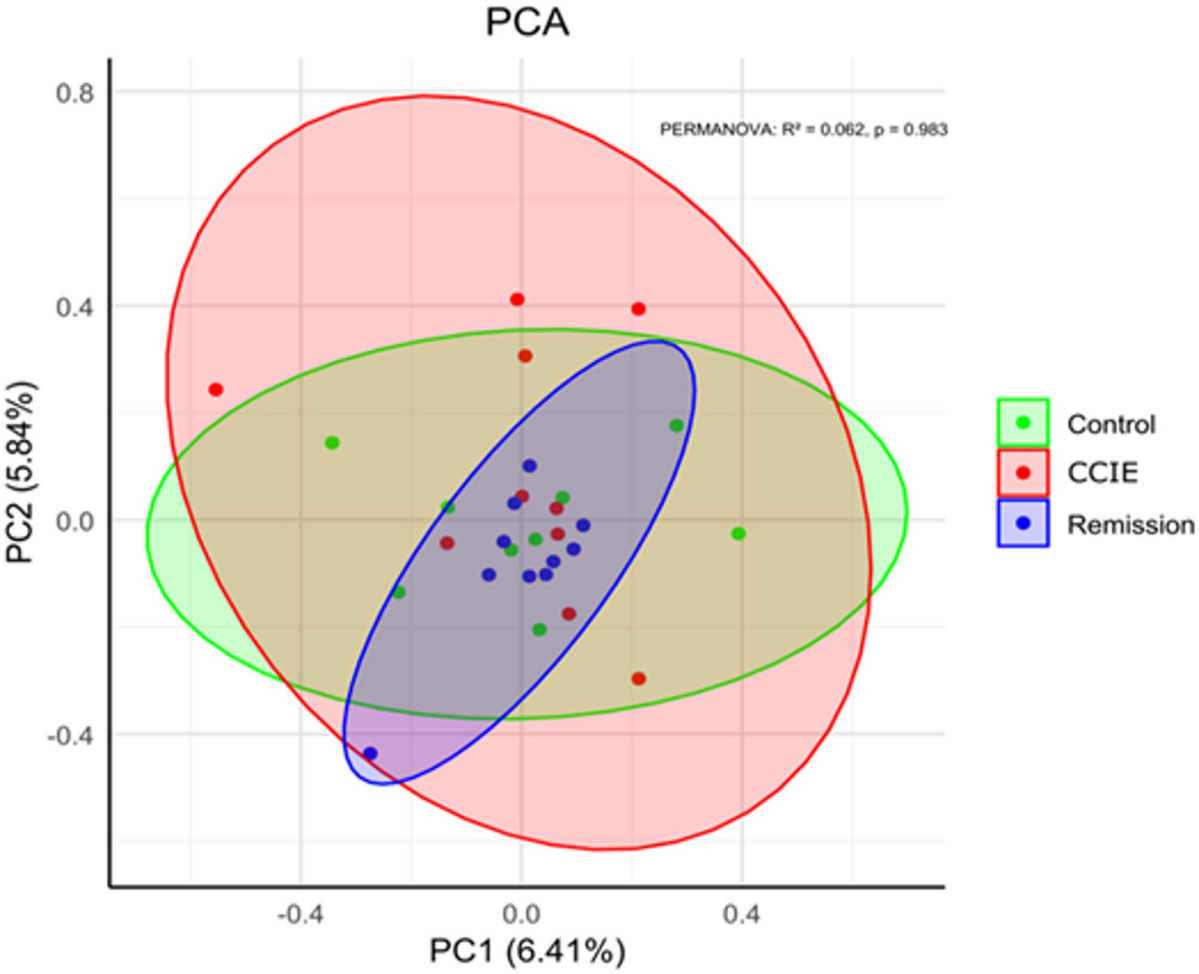
Principal Component Analysis of the plasma proteome of patients with CCIE during active disease (CCIE) and clinical remission (Remission) compared to healthy controls (Control). There is no statistically significant separation between the three groups in terms of PCA Clustering.

**Fig. 5:**
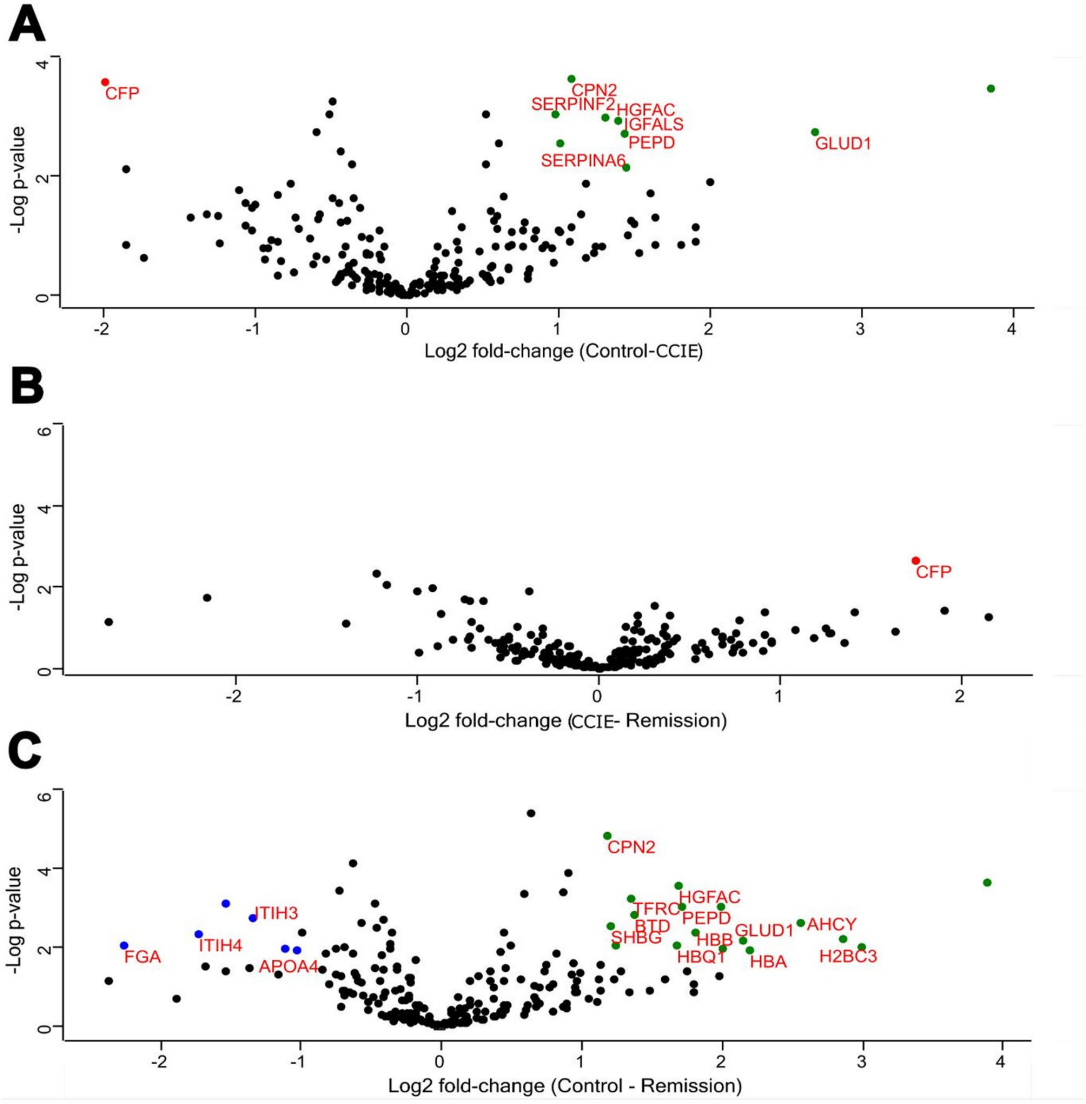
Volcano plots (− log p-values versus log2 fold-change) of the intensity of plasma proteins of from the three conditions in this study (**A**–**C**). Proteins with a minimum 2-fold intensity change compared to the control (log2 fold-change ≥ 1 or log2 fold-change ≤ − 1) and a q-value ≤ 0.05 were considered significantly abundant. Black dots represent non-significant differentially abundant proteins, red dots show the significantly higher abundance proteins in the CCIE group, green dots the significantly higher abundance proteins in the control group and the blue ones represent the significantly higher abundance proteins in the remission group.

Initially 465 proteins were identified using LC-MS. After removing proteins only identified by site, reverse hits, and potential contaminants, 435 proteins remained. Subsequently, we excluded proteins that were identified in less than 3 samples in at least one group (CCIE, Remission, Control). The above filtering process resulted in 222 proteins being available for further analysis. The identified proteins across all samples and all raw data are available in the supplementary material (Supplementary Table 1). The ranking of protein abundance can be observed in Fig. 3. All proteins were identified amongst all samples of all 3 groups, as no exclusivity was detected. The principal component analysis (PCA) revealed no separation between the three groups in terms of PCA clustering (Permanova R^2^ = 0.062, *p* > 0.5) (Fig. 4). Firstly, we compared the plasma proteome of healthy controls to dogs with CCIE during enrollment. Patients in the CCIE group had two times higher log2 fold-change of plasma complement factor properdin (CFP) when compared to controls (log2 fold-change = 1.98, *q = 0.012*). On the other hand, eight (*N* = 8) proteins, like hepatocyte growth factor activator (HGFAC), insulin-like growth factor binding protein (IGFALS), carboxypeptidase N subunit 2 (CPN2), transcortin (SERPINA6), peptidase D (PEPD), glutamate dehydrogenase 1 (GLUD1), Serpin family F member 2 (SERPINF2) and Ig-like domain-containing protein (A0A8I3PVB5) were significantly less abundant in the proteome of the CCIE group (Fig. 5A). Of these proteins, A0A8I3PVB5 was the most significantly downregulated protein in the RG (fold change= −3.8, *q = 0.014*). When the patients of the CCIE group entered clinical remission as defined in the materials and methods, they were changed to the RG and the plasma proteome of the two sampling points were compared. Interestingly, there was only one protein in either group that showed significant differential abundance, as the two proteomes show close similarity (Fig. 5B). The protein that was differentially more abundant in the proteome of the CCIE dogs compared to their plasma proteome after entering clinical remission and joining the RG was again CFP (log2 fold-change 1.74 *q = 0.036*). At the same time, the comparison of the proteome of the dogs during clinical remission with that of the healthy controls demonstrated further significant differences. Seven (*N* = 7) proteins were differentially more abundant in the RG. These proteins were inter-alpha-trypsin inhibitor heavy chain 3 (ITIH3), inter-alpha-trypsin inhibitor heavy chain 4 (ITIH4), fibrinogen alpha chain (FGA), transthyretin (TTR), apolipoprotein 4 (APOA4) and 2 Ig-like domain-containing proteins (A0A8I3S5N2 and A0A8I3PB96). Of these proteins FGA was the most differentially abundant protein of the RG proteome (log2 fold-change 2.26, q = 0.02). On the other side, the expression of several proteins was downregulated in comparison to the control proteome (*N* = 18). Of these, the most notable were again HGFAC, carboxypeptidase N subunit 2 (CPN2) (highest log2 fold-change − 2 *q < 0.01*), transferrin receptor protein (TFRC), histone H2B (H2BC3), adenosylhomocysteinase (AHCY) and glutamate dehydrogenase 1 (GLUD1) (Fig. 5C).

**Fig. 6:**
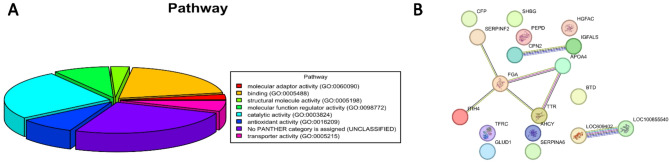
(**A**, **B**). (**A**) Gene Ontology enrichment analysis of the differentially abundant plasma proteins from dogs with CCIE during enrollment and after reaching clinical remission. Only genes that had a functional category described are represented. (**B**)The string network figure illustrates the interactions between various plasma proteins of the dogs with chronic enteropathies during both sampling points (enrollment and remission). The significant FDR-adjusted p-value was used as including criterion with regard of the expression level. Each node represents a unique protein, and the edges connecting nodes indicate known or predicted interactions. Node color indicates the type of interaction evidence, ranging from experimental data to curated databases and text mining.

The quantified proteins were used to perform gene ontology enrichment analysis using the Panther 19.0 software^46^, to investigate a potential functional significance of the differentially abundant proteins between patients in the dogs suffering from CCIE at both sampling points and healthy dogs (Fig. 6A). The molecular pathways involve immune system pathways like complement activation, antioxidant activity including glutamate (GLUD1) and reactive oxygen species metabolism, inflammatory response, coagulation cascade and hormone and transport pathways. Additionally, the network of proteins and their interaction was illustrated to demonstrate the direct and indirect associating between differentially abundant proteins. This method clusters proteins considering multiple parameters that includes physical interaction, gene vicinity, functional relationship among others^47^. The interconnectivity of the up-regulated and down-regulated plasma proteins of the dogs with chronic enteropathy is illustrated in Fig. 6B.

## Discussion

In our study, we analyzed the plasma proteome of dogs with histologically confirmed CCIE and compared it to the plasma proteome of the same dogs during remission and to that of healthy controls. The amount of identified proteins is similar to that reported in previous studies^37,48,49^. The plasma proteome of the healthy dogs in the control group was similar to this reported in our previous study without significant alterations^37^. In accordance with the literature, intestinal chronic inflammation was significantly associated with nutritional deficits resulting in lower total protein, albumin, and bioavailable cobalamin^1,11,42,50^. Moreover, we were able to identify proteins, whose plasma concentration is significantly altered in dogs with CCIE. In our cohort, particularly plasma complement factor properdin (CFP) stood out, showing 2 times higher log2 fold-changes in dogs of the CCIE group compared to healthy controls. CFP, also named properdin, is a positive regulator of the alternative pathway of the complement system^51^. It binds to many microbial surfaces and apoptotic cells and stabilizes the C3- and C5-convertase enzyme complexes in a feedback loop that ultimately leads to formation of the membrane attack complex and lysis of the microbe/cell^51^. As such, greater levels of CFP imply an ongoing battle/inflammation against the residing microbes, that even remained in patients who reached clinical remission. CFP may therefore be suitable marker indicating subclinical chronic intestinal inflammation. It must be emphasized that this protein was the sole differentially abundant protein to have a significant log2 fold-change in the CCIE group vs. RG. Specific studies investigating the role of plasma CFP in canine CCIE are currently lacking. In humans, alterations in properdin levels have been linked to various inflammatory and autoimmune conditions^52^. A study in people reported that CFP increased during active phases of ulcerative colitis and Crohn’s disease^53^ and remained high also when individuals went into remission^53^ which is in accordance with our results in dogs. In contrast, in another study, human patients with both Crohn’s disease and ulcerative colitis with extraintestinal complications exhibited reduced serum CFP levels compared to healthy controls^54,55^, which may be indicative that once extraintestinal complication arise the contribution of the gut-specific immune response becomes smaller.

**Fig. 7:**
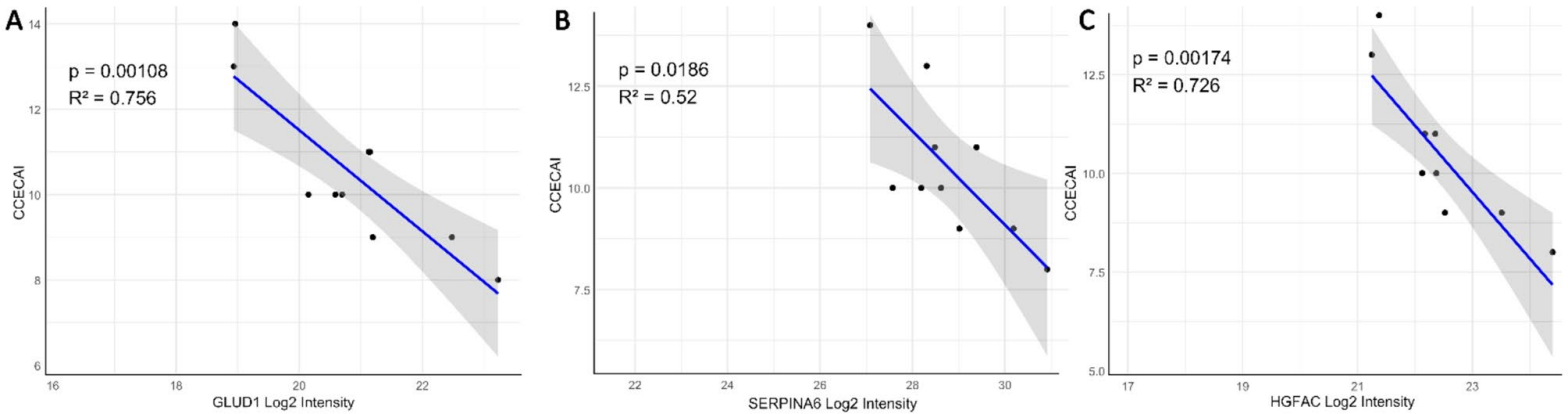
(**A**, **B**, **C**). Linear regression analysis of the log2 intensity of the glutamate dehydrogenase 1 (GLUD1) (**A**), transcortin (SERPINA6) (**B**), and hepatocyte growth factor activator (HGFAC) (**C**) and the canine chronic enteropathy activity index (CCECAI). There is a significant negative correlation between GLUD1, HGFAC and transcortin levels and disease severity reflected by the CCECAI, as GLUD1, HGFAC and SERPINA6 concentrations linearly decrease with increased disease severity.

In contrast, eight proteins were significantly downregulated in the plasma of the CCIE group compared to the healthy dogs. GLUD1, a mitochondrial glutamate dehydrogenase, an enzyme important in regulating amino acid-induced insulin secretion, was significantly downregulated in dogs with CCIE compared to healthy controls. This may be due to the lower nutritional bioavailability of glutamate due to malabsorption and inflammation^56–58^ that results in lower levels of GLUD1. GLUD1 was negatively correlated to disease severity (r^2^ = 0.756 *p* = 0.0018) (Fig. 7A).

SERPINA6 (transcortin), acts as glucocorticoid binding protein and is a negative acute phase protein. It releases cortisol upon pyrexia and acidemia or neutrophil elastase cleavage to body sites when cortisol is required^59^. It is known that its expression is downregulated in the liver by increased systemic levels of inflammatory cytokines that result in an increase in the free serum cortisol^60,61^. Consequently, the significantly lower plasma levels of transcortin in dogs with CCIE reflect on one hand inflammation and on the other hand the ongoing regulatory mechanism in patients with CCIE. There may also be an association with disease severity similarly to humans^62^. This hypothesis is strengthened by the fact that in our CCIE cohort, the negative correlation between SERPINA6 log2 intensity and disease severity (CCECAI) was significant (Pearson r^2^ = 0.54, *p* = 0.018) (Fig. 7B).

HGFAC is a serine protease responsible for converting inactive pro-Hepatocyte Growth Factor into its active form HGF. Active HGF plays a significant role in tissue regeneration and repair by promoting cell proliferation, motility, and morphogenesis^62^. Recent research has identified HGFAC as a potential therapeutic target in human IBD, but no specific treatment has yet been tested^63,64^. In this study, HGFAC was associated with reduced risk of IBD, and modulating HGFAC activity is speculated to influence IBD progression or severity^64^. Our study is the first to show the significant downregulation of HGFAC in dogs with CCIE, both during active disease and remission. Similarly to SERPINA6 and GLUD1, HGFAC showed significant negative correlation with disease severity (Pearson r^2^ = 0.726 *p* = 0.0017) (Fig. 7C). As canine and human CCIE share many similarities in terms of pathophysiology and clinical presentation, it is plausible that HGFAC could play a comparable role in dogs. Further research is needed to elucidate HGFAC’s involvement in canine CCIE and to assess its potential as a therapeutic target.

IGFALS is part of the insulin-like growth factor system, which is important for growth, development and regeneration^65^, which lower levels consistently reported upon chronic inflammatory conditions^66^ and patients with IBD^67^.

Additionally, we compared the plasma proteome of dogs entering clinical remission as defined above with the plasma proteome of healthy controls. Here, our network analysis particularly linked FGA, TTR, APO4 as well as ITIH4 together, which were all upregulated compared to control in patients on remission.

Briefly, the calcium-binding fibrinogen alpha chain (FGA), though a major component of blood clot formation, has an additional function during the early stages of wound repair to stabilize lesions and guide cell migration during re-epithelialization.

Inter-alpha-trypsin inhibitor heavy chain 4 (ITIH4) is an acute phase protein usually involved in inflammatory responses to trauma. ITIH4 and ITIH3 play a role in inflammation and autoimmune diseases, and their concentration has been reported higher in people with IBD. This suggests an association with disease activity rather than achieving clinical remission^68^.

Transthyretin is considered a negative acute phase protein, which transports the thyroid hormone, and is also crucially involved in retinol transport. It is implicated in nerve regeneration and glucose homeostasis. Here, an increased concentration likely indicates the greater dietary demand of Vitamin A in the remission phase.

Furthermore, APOA4, a protein which is the major component of HDL and chylomicrons, is involved in lipid metabolism and considered anti-inflammatory. Here decreased levels have been reported in people with IBD, which normalizes during remission^69^. In our study APOA4 concentration in the RG-group was not significantly different from the CCIE group but was elevated compared to healthy dogs. Our interpretation dictates that while ongoing (sub-) inflammation is present, simultaneously also repair mechanism are in place. The elevated levels of APO4 may also indicate greater chylomicron formation to overcome the presence of the mucosal block for micronutrients^58^.

Most of the differentially abundant proteins identified are involved in the acute phase response and pathways like DNA modeling, coagulation, histone modifications, chromatin remodeling and methylation^70,71^. Hence, our data clearly emphasize that subclinical inflammation is still present even during remission with elevated CFP being here indicative, while negative acute phase proteins such as transcortin, HGFAC and IGFALS were downregulated and are also linked to disease severity.

Our study has several limitations. Firstly, the small cohort size limits the applicability of the findings to the broader CCIE population, as it may not capture the full spectrum of variability. This also raises concerns about potential overrepresentation or underrepresentation of specific subgroups, for example underrepresentation of dogs with severe CCIE and PLE. Future studies with larger and more diverse populations are necessary to validate these findings and enhance their applicability. To reduce cost pooling disease subtypes to focus on disease patterns would be one strategy to limit cost, but expand our knowledge as we previously demonstrated^37,49^. Another limitation is the narrow protein-range and identification criteria we applied in this study, to ensure reliable results. In future studies, expanding the protein panel would enable the identification of a greater number of proteins in the disease progression. During data analysis, we apply strict identification criteria and differentially abundant proteins that were not identified in at least 3 patients of each group were excluded from further analysis to ensure homogeneity. This resulted in many proteins that were significantly different in abundance in dogs with more severe CCIE and the PLE dog to be excluded from the statistical analysis. Many significant differences were noted when comparing dogs with mild to moderate CCIE to dogs with severe CCIE or PLE, but as the small number of animals in the subgroup, no conclusions can be made, and a larger sample size is needed in future studies. Therefore, we herein report fewer significantly differentially abundant proteins between CCIE and control group as expected.

An additional limitation arises from the noticeable variability in body weight, age, and breed within groups and between CCIE and healthy dogs. These differences can influence the outcomes in ways that are unrelated to our primary research question, potentially leading to skewed or biased results. This limitation can also be addressed by sample pooling. Furthermore, sampling and analysis on more time periods and during longer remission might reveal more significant alterations in protein abundance. Therefore, while the study provides valuable insights, these limitations should be considered when drawing conclusions or extrapolating the findings to a larger and more diverse population.

## Conclusion

Our study clearly demonstrates that even during remission subclinical inflammation is present in CCIE patients. Properdin (CFP) linked the disease etiology also to microbes and could arise as a potential biomarker to detect subclinical inflammation. We also present HGFAC as a potential protein associated with decreased risk for CCIE, which could serve as a potential therapeutic target, similarly to recent data in human medicine. We confirmed the role of SERPINA6 as negative phase protein, that demonstrates linearly decreased concentration with increased disease severity. Finally, proteins such as APOA4 and ITIH3/4 could be promising as indicators of remission. These findings underscore the potential of plasma proteomic analysis in improving the diagnosis and management of canine chronic enteropathies. Further studies with larger cohorts are essential to validate these biomarkers and advance individualized diagnostic approaches.

## Electronic supplementary material

Below is the link to the electronic supplementary material.

Supplementary Material 1

## Data Availability

All data generated or analysed during this study are included in this published article [and its supplementary information files].
